# A new HPLC method with multiple detection systems for impurity analysis and discrimination of natural versus synthetic cannabidiol

**DOI:** 10.1007/s00216-024-05396-5

**Published:** 2024-06-28

**Authors:** Virginia Brighenti, Matilde Marani, Clarissa Caroli, Laura Bertarini, Alessio Gaggiotti, Federica Pollastro, Caterina Durante, Giuseppe Cannazza, Federica Pellati

**Affiliations:** 1 Department of Life Sciences, University of Modena and Reggio Emilia, Via G. Campi 103, Modena, 41125 Italy; 2 Clinical and Experimental Medicine PhD Program, University of Modena and Reggio, Via G. Campi 287, Modena, 41125 Italy; 3 Farmech, Piazza Duomo 20, Milan, 20122 Italy; 4 Department of Pharmaceutical Sciences, University of Eastern Piedmont, Largo Donegani 2, Novara, 28100 Italy; 5 Department of Chemical and Geological Sciences, University of Modena and Reggio Emilia, Via G. Campi 103, Modena, 41125 Italy

**Keywords:** Cannabidiol, Origin, Impurity, HPLC, HRMS, Chiral analysis

## Abstract

Cannabidiol (CBD) is the main non-psychoactive phytocannabinoid derived from *Cannabis sativa* L. It is now an active pharmaceutical ingredient (API), given its usage in treating some types of pediatric epilepsy. For this reason, this compound requires a deep characterization in terms of purity and origin. Previous research work has shown two impurities in CBD samples from hemp inflorescences, namely, cannabidivarin (CBDV) and cannabidibutol (CBDB), while abnormal-cannabidiol (abn-CBD) has been described as the primary by-product that is generated from CBD synthesis. Both natural and synthetic CBD samples exhibit the presence of Δ^9^-tetrahydrocannabinol (Δ^9^-THC) and Δ^8^-THC. This study aimed to develop a new analytical method based on high-performance liquid chromatography (HPLC) with different detection systems to study the purity of CBD and to define its origin based on the impurity profile. In addition to the above-mentioned cannabinoids, other compounds, such as cannabigerovarin (CBGV), cannabigerol (CBG), cannabichromevarin (CBCV), and cannabichromene (CBC), were examined as potential discriminating impurities. Qualitative and quantitative analyses were carried out by UHPLC-HRMS and HPLC-UV/Vis, respectively. Principal component analysis was applied for statistical exploration. Natural CBD samples exhibited purities ranging between 97.5 and 99.7%, while synthetic samples were generally pure, except for three initially labeled as synthetic, revealing natural-derived impurities. To further confirm the origin of CBD samples, the presence of other two minor impurities, namely cannabidihexol (CBDH) and cannabidiphorol (CBDP), was assessed as unequivocal for a natural origin. Finally, an enantioselective HPLC analysis was carried out and the results confirmed the presence of the (–)-*trans* enantiomer in all CBD samples. In conclusion, the HPLC method developed represents a reliable tool for detecting CBD impurities, thus providing a clear discrimination of the compound origin.

**Graphical Abstract:**

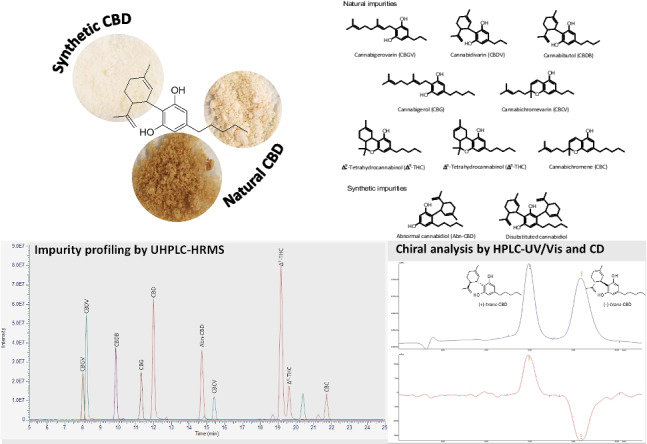

**Supplementary Information:**

The online version contains supplementary material available at 10.1007/s00216-024-05396-5.

## Introduction

Cannabidiol (CBD) is one of the main non-psychoactive phytocannabinoids produced by the well-known plant *Cannabis sativa* L. and, in particular, it is the most abundant compound in the inflorescences from fiber-type varieties (also known as hemp) [1, 2]. It is a molecule with high value in the pharmaceutical field, given its wide spectrum of potential therapeutic effects and its favorable safety profile [3].

CBD has established anti-seizure effects, which are confirmed by its approval for treating certain types of epilepsy [4]. Indeed, it is marketed as an oral solution under the name Epidiolex^®^ as a monotherapy for the treatment of children rare and severe forms of seizure, such as Lennox-Gastaut and Dravet syndromes [4]. Sativex^®^ is another commercial formulation containing CBD together with ∆^9^-tetrahydrocannabinol (∆^9^-THC), and it is used in multiple sclerosis to relieve symptoms of muscle stiffness (spasticity) [5].

Notably, CBD exhibits antioxidant, anti-inflammatory, and neuroprotective properties, which make it a promising candidate for managing different pathologies [3, 6–8]. Additional roles, including its potential anti-proliferative properties against cancer cells, are deeply investigated in the ongoing research [9].

CBD as a pure substance can be obtained either by extraction from hemp inflorescences and subsequent purification [10] or by organic synthesis [11]. Prior to the extraction step, the plant material is usually subjected to heating to convert cannabinoid acids into their neutral counterparts [10]. Among the extraction techniques, maceration of the plant material in an organic solvent is the most widely used procedure, due to its simplicity. Ethanol (EtOH) represents the solvent of choice, but supercritical fluid extraction (SFE) with carbon dioxide (CO_2_) is also frequently employed [12]. After the extraction, the crude extract can be further purified by preparative liquid chromatography [13].

As an alternative to the extraction of the plant material, CBD can be easily obtained by means of organic synthesis, with the main challenge being the stereoselective synthesis of (−)-*trans*-CBD, the only isomer found in nature [11]. Among the different synthetic approaches described in the literature, the shortest one for the stereoselective synthesis of CBD is based on a single-step reaction involving a Friedel-Crafts allenylation [11]. This reaction utilizes readily available olivetol and (+)-*p*-mentha-2,8-dien-1-ol as the chiral electrophile precursor in the presence of acid catalysis. The reaction results in the formation of two secondary products, such as abnormal-CBD (abn-CBD) and dialkylated olivetol (or disubstituted-CBD), a compound that undergoes two acid-catalyzed condensation reactions [11].

It should be pointed out that, as described in the literature, the way CBD is obtained does not affect its biological activity, since both natural and synthetic CBD display the same biological effects *in vitro* [14]. However, CBD obtained by the aforementioned routes can greatly differ, being the isolation from a natural raw extract more challenging with respect to the purification from a reaction mixture. Therefore, it is of outmost importance to discriminate natural-derived CBD from the synthetic one to have a reliable and correct classification of the substance, especially when it is used as an active pharmaceutical ingredient (API) for the preparation of medicinal products [3]. This issue becomes more crucial by considering the local regulations of CBD in different countries. Usually, preparations containing predominantly CBD and less than 0.2–0.3% of ∆^9^-THC are not under international control [15, 16].

In this perspective, the development of reliable analytical methods able to determine CBD purity and correctly classify the origin (natural or synthetic) of the samples plays a pivotal role to ensure the appropriate pharmaceutical characterization of this API. To do this, it is necessary to develop efficient methodologies that allow for the unequivocal identification of natural CBD from that produced using synthetic strategies.

**Fig. 1:**
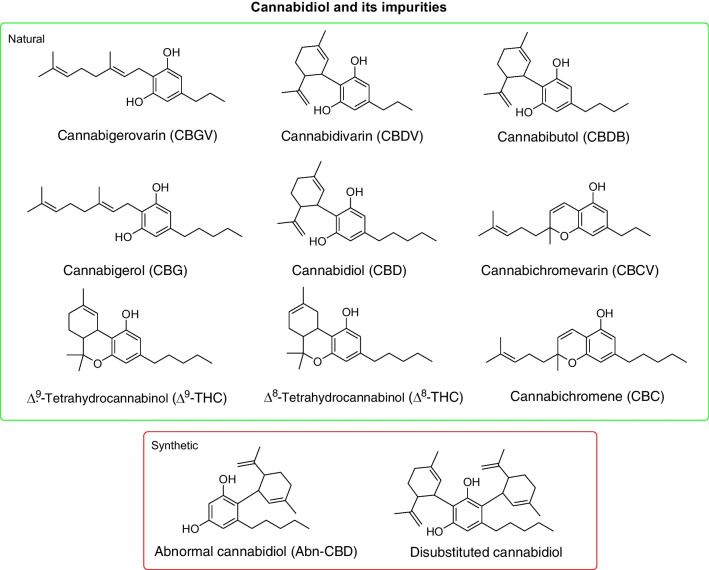
Chemical structures of cannabidiol (CBD) and related natural and synthetic impurities

In the literature, the discrimination of CBD origin is mainly based on the analysis of its impurity profile using high-performance liquid chromatography (HPLC) [17, 18]. This is the most frequently applied technique for the analysis of cannabinoids in different matrices, including the plant material, its derived products, and biological fluids [17, 18]. In detail, target impurities for natural CBD include other cannabinoids derived from the extraction of the plant material subjected to a heating process to obtain only decarboxylated cannabinoids, such as those structurally related to CBD, including cannabidivarin (CBDV) and cannabibutol (CBDB) (Fig. 1) [19]. Δ^9^-THC can also be detected in natural-derived CBD samples, representing another possible impurity (Fig. 1) [20]. Regarding synthetic CBD, despite conducting stereoselective syntheses, by-products can be obtained, such as abn-CBD and dialkylated olivetol (or disubstituted-CBD) (Fig. 1) [20], even though these impurities are usually later removed through chromatographic purification [20]. From the literature, it is also evident that, even after a synthetic process, traces of Δ^9^-THC and Δ^8^-THC may be present, since CO_2_ and water (H_2_O) from the air can create the acidic conditions responsible for CBD cyclization [20].

Another strategy for the authentication of natural-derived CBD involved the stable isotope ratio analysis [21]. Indeed, the analysis of the stable isotope ratios of oxygen and hydrogen was useful in the discrimination of CBD of a totally natural origin from that obtained through chemical synthesis [21]. The identification of adulterated CBD products obtained by mixing natural and synthetic CBD was found to be difficult [21].

**Fig. 2:**
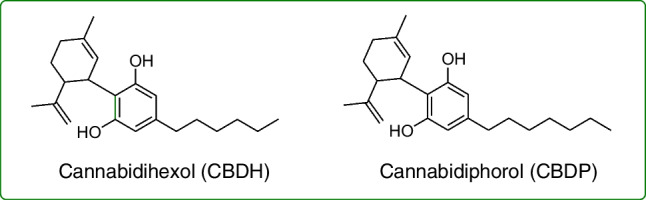
Chemical structures of CBD homologues cannabidihexol (CBDH) and cannabidiphorol (CBDP)

In the light of all the above, the aim of this work was to develop a method for multi-component separation, based on HPLC with different detection systems, for the purity assessment of CBD and the reliable classification of the sample origin based on the cannabinoid profiles. In addition to ∆^9^-THC, ∆^8^-THC, CBDV, and CBDB, the cannabinoids investigated as putative discriminating compounds in CBD samples included cannabigerovarin (CBGV), cannabigerol (CBG), cannabichromevarin (CBCV), and cannabichromene (CBC) (Fig. 1). To confirm the origin of some uncertain samples, two CBD homologues having a different length of the side chain, i.e., cannabidihexol (CBDH) and cannabidiphorol (CBDP), were analyzed for the first time as unequivocal identifiers of a natural origin, as they are minor cannabinoids deriving from the plant material only (Fig. 2) [22, 23]. All CBD samples were also assessed to evaluate their enantiomeric excess, using chiral HPLC with circular dichroism (CD).

## Materials and methods

### Chemicals

Methanol (MeOH), acetonitrile (ACN), and formic acid (HCOOH) were purchased from Sigma-Aldrich (Milan, Italy), while ammonium formate was from Fluka (Charlotte, NC, USA). H_2_O was purified using a Milli-Q^®^ Advantage 10 system from Millipore (Milan, Italy).

### Standard compounds

The standard solution of CBG (1 mg/mL in MeOH) was purchased from Restek (Milan, Italy). Abn-CBD reference solution (1 mg/mL in ethyl acetate) was from Santa Cruz Biotechnology (Dallas, TX, USA). Reference compounds of CBGV, CBDV, CBCV, and CBC were kindly provided by Prof. Federica Pollastro of the Department of Pharmaceutical Sciences of the University of Eastern Piedmont (Italy), while reference solutions of CBDB (100 μg/mL in MeOH), CBDH and CBDP (1 mg/mL in MeOH) were provided by Prof. Giuseppe Cannazza of the Department of Life Sciences of the University of Modena and Reggio Emilia (Italy).

### CBD samples

Twenty-seven samples of CBD powder were analyzed in this study, obtained both by extraction from hemp inflorescences, from here on referred to as natural CBD (Nat1-18), and by organic synthesis, from here on referred to as synthetic CBD (Syn1–9). All the samples were provided by Farmech (Milan, Italy), with the only exception of sample Syn6, which was synthesized at the Department of Life Sciences of the University of Modena and Reggio Emilia, and sample Nat18, which was purchased from Cerilliant (Round Rock, TX, USA). These two samples were used as reference for synthetic and natural CBD, respectively.

### Sample preparation for UHPLC-HRMS and HPLC-UV/Vis analysis

The stock solutions of CBD samples were prepared by weighing an appropriate amount of compound and dissolving it in MeOH to reach the final concentration of 1 mg/mL. Then, the solution was diluted with MeOH to obtain a 200 µg/mL concentration. The test solution was finally filtered through a 0.45 μm polytetrafluoroethylene (PTFE) filter into the HPLC vial. The sample preparation was performed in duplicate for each sample and stored at the temperature of 4 °C until analysis was performed.

### UHPLC-HRMS analysis

UHPLC-HRMS analyses were performed on a Thermo Scientific (MA, USA) UHPLC Ultimate 3000 system, equipped with a vacuum degasser, a binary pump, a thermostatted autosampler, a thermostated column compartment, and a Q-Exactive Orbitrap mass spectrometer with a heated electro-spray ionization (HESI) source. The separation of compounds was achieved on an Ascentis Express C_18_ column (150 mm × 3.0 mm I.D., 2.7 µm, Supelco, Bellefonte, PA, USA) chromatographic column with a mobile phase composed of a 2 mM ammonium formate solution in H_2_O with 0.1% HCOOH (A) and 0.1% HCOOH in ACN (B). A gradient elution was performed as follows: 0–20 min from 70 to 90% B, which was held for 5 min; the post-running time was set at 10 min. Flow rate and injection volume were set at 0.2 mL/min and 3 µL, respectively.

As for MS acquisition, the HESI source was operated both in the positive and the negative ion modes. The MS source parameters were set as follows: sheath gas (N_2_) 30, auxiliary gas (N_2_) 20, auxiliary gas temperature 290 °C, electro-spray voltage 3.5 kV (+) and 3.0 kV (–). The analyses were acquired in the full mass data-dependent (FM-dd-MS/MS) mode at a resolving power of 35.000 full width at half maximum (FWHM). The other mass analyzer parameters were set as follows: scan range *m*/*z* 200–1000, automatic grain control (AGC) target 1×10^6^ ions in the Orbitrap analyzer, ion injection time 200 ms, and isolation window for the filtration of the precursor ions *m*/*z* 1.0. The fragmentation of precursor ions was performed at 28% and 70%, as normalized collision energies (NCE). The NCE principle automatically compensates for the mass dependency of the collision energy needed to achieve the optimum fragmentation efficiency.

### HPLC-UV/Vis analysis

HPLC-UV/Vis analyses were performed on an Agilent Technologies (Waldbronn, Germany) modular model 1260 Infinity II system, consisting of a quaternary pump, a manual injector, and a UV variable wavelength detector. Chromatograms were recorded by using an Agilent OpenLab ChemStation (Rev. C.01.10). Chromatographic parameters were the same as those applied to the UHPLC-HRMS detection. Chromatograms were recorded at the wavelength of 210 nm for the detection of selected cannabinoids. Due to the high concentration of CBD in solution, as required for the impurity characterization, a semi-quantitative analysis was performed, based on the ratio between the peak area of the compound (CBD and impurities) and the total chromatogram peak area was calculated as percentage (%) relative peak area. The level of impurities was determined using a % relative peak area ≥0.05. Each sample was analyzed in duplicate with two injections for each solution. Data are therefore the mean of four results.

### Multivariate analysis

The results from HPLC-UV/Vis were arranged in a bi-dimesional matrix with samples on the row and investigated variables on the column. The % relative peak areas of CBD and its main impurities (CBDV and CBDB) were considered the variables for the multivariate analysis. Data were auto-scaled and principal component analysis (PCA) was carried out by using PLS_Toolbox 9.2.1 software (Eigenvector Research Inc., Manson, WA, USA) for MATLAB®.

### Enantioselective HPLC analysis

The enantioselective HPLC analysis on CBD was carried out on a CHIRALPAK AD-RH [amylose tris (3,5-dimethylphenylcarbamate)] (150 × 4.6 mm I.D., 5 µm) (Chiral Technologies Europe S.A.S, France) based on amylose with a mobile phase composed of 60% ACN (as organic modifier) and 40% of 0.1% HCOOH. An isocratic elution was followed with a flow rate of 1.5 mL, with an injection volume of 10 μL. The detection was performed on a Jasco CD-2095 Plus chiral detector interfaced to the HPLC system through a JMBS Hercule Lite interface (Jasco Europe, Italy). The detector operated at the wavelength of 228 nm. Chromatograms were acquired online and processed using Borwin software 1.5 (Jasco Europe, Italy).

## Results and discussion

### Optimization of the UHPLC-HRMS method for CBD impurity analysis

The aim of this work was to optimize a new HPLC method, using different detection systems, that can be useful to determine the purity of CBD and discriminate its origin (i.e., natural or synthetic) by the profiling of its impurities. Before proceeding with the analysis of real samples, the analytical method optimization was performed. A mixture of cannabinoid reference compounds, including the most representative possible impurities of both natural and synthetic CBD (CBGV, CBDV, CBDB, CBG, CBD, abn-CBD, CBCV, Δ^9^-THC, Δ^8^-THC, and CBC) (Fig. 1), was prepared and injected into the UHPLC system at a concentration of 50 μg/mL. Acidic cannabinoids were not considered in the method optimization as, in the case of extraction from hemp inflorescences, the plant material is submitted to high temperatures to convert cannabinoid acids into the neutral ones, prior to the extraction process [17, 18]. Disubstituted-CBD was also not included in the pool of possible impurities, since the preliminary HRMS data analysis allowed us to exclude its presence in the samples.

By applying the literature parameters, the right separation between CBGV and CBDV was not completely achieved [24]. This issue was overcome thanks to the usage of an Orbitrap MS analyzer, given the different molecular weights (MW) of the two analytes. The choice of using the UHPLC-HRMS technique relied on high accuracy and precision in the determination of the exact mass and fragmentation spectra of the analytes [19]. In addition, the flow rate was adjusted at 0.2 mL/min to get a better separation of CBGV and CBDV. The mass analyzer was operated in the positive ion mode, given the better ionization of the analytes.

**Fig. 3:**
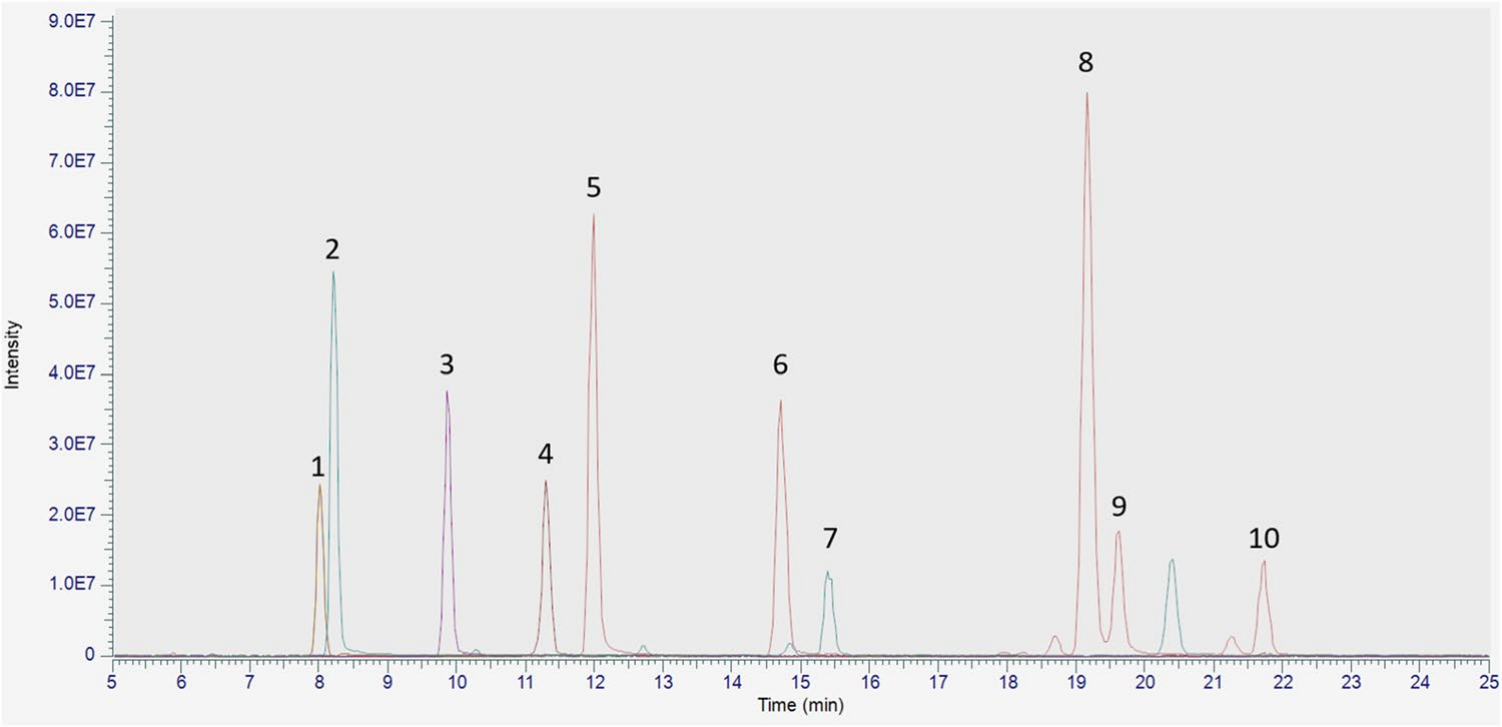
Extracted ion chromatogram (EIC) from the UHPLC-HRMS analysis of the mixture of reference cannabinoids. For peak identification, see Table 1

The retention times (*t*_R_) and the precursor and product ions for each compound considered in the present study are listed in Table 1, while the extracted ion chromatogram (EIC) from the UHPLC-HRMS analysis of the cannabinoid mixture is shown in Fig. 3. It is possible to observe that the target compounds share a common fragmentation pattern: indeed, the main product ion generated by all neutral cannabinoids was due to the loss of a fragment corresponding to the terpenic moiety (−122 Da). The only exception to this fragmentation behavior was represented by abn-CBD, which showed a main product ion at *m/z* 221.1531 (− 94 Da).

**Table 1:** Chromatographic and spectrometric data of CBD and its impurities (chromatographic parameters are described in the “Materials and methods” section)

Peak n.	Compound	*t*_R_ (min)	Acquisition mode	Precursor ion (*m/z*)	Product ion (*m/z*)
1	CBGV	8.0	+	289.2154	165.0907 (100) 123.0439 (25) 95.0494 (5) 81.0703 (4) 69.0704 (3) 67.0548 (6)
2	CBDV	8.2	+	287.1998	287.1998 (100) 231.1375 (25) 165.0907 (60) 135.1166 (25) 123.0440 (35) 93.0702 (28)
3	CBDB	9.9	+	301.2154	301.2154 (100) 245.1532 (23) 179.1063 (52) 123.0440 (36) 93.0702 (29) 81.0703 (24)
4	CBG	11.3	+	317.2467	193.1220 (100) 137.0595 (4) 123.0440 (30) 95.0494 (5) 81.0703 (5) 67.0548 (4)
5	CBD	12.0	+	315.2310	315.2310 (100) 259.1686 (28) 193.1219 (50) 135.1165 (27) 123.0440 (39) 93.0702 (29)
6	abn-CBD	14.7	+	315.2310	315.2310 (53) 221.1531 (100) 135.1165 (40) 107.0856 (34) 93.0702 (84) 91.0545 (22)
7	CBCV	15.4	+	287.1998	287.1997 (28) 231.1373 (32) 165.0907 (100) 123.0440 (22) 81.0703 (58) 69.0705 (31)
8	Δ^9^-THC	19.2	+	315.2310	315.2310 (100) 259.1687 (28) 193.1219 (52) 123.0440 (45) 93.0701 (34) 81.0703 (30)
9	Δ^8^-THC	19.6	+	315.2310	315.2310 (100) 259.1685 (27) 193.1218 (42) 135.1165 (28) 123.0439 (36) 93.0701 (28)
10	CBC	21.8	+	315.2310	315.2310 (27) 259.1685 (42) 193.1218 (100) 123.0439 (30) 81.0703 (67) 69.0704 (29)

Twenty-seven samples of pure CBD were analyzed in this work by UHPLC-HRMS, encompassing seventeen obtained by extraction from *C. sativa* inflorescences (natural CBD) and eight by organic synthesis (synthetic CBD), as specified by the sample supplier. Additionally, sample Syn6 served as a control for synthetic CBD, while sample Nat18 was the control for natural CBD. By applying the UHPLC-HRMS method to CBD samples, it was possible to qualitatively identify the impurities present within them. Impurities were identified by comparing *t*_R_, exact mass values and fragmentation spectra of the analytes in the samples with those of pure reference compounds.

**Fig. 4:**
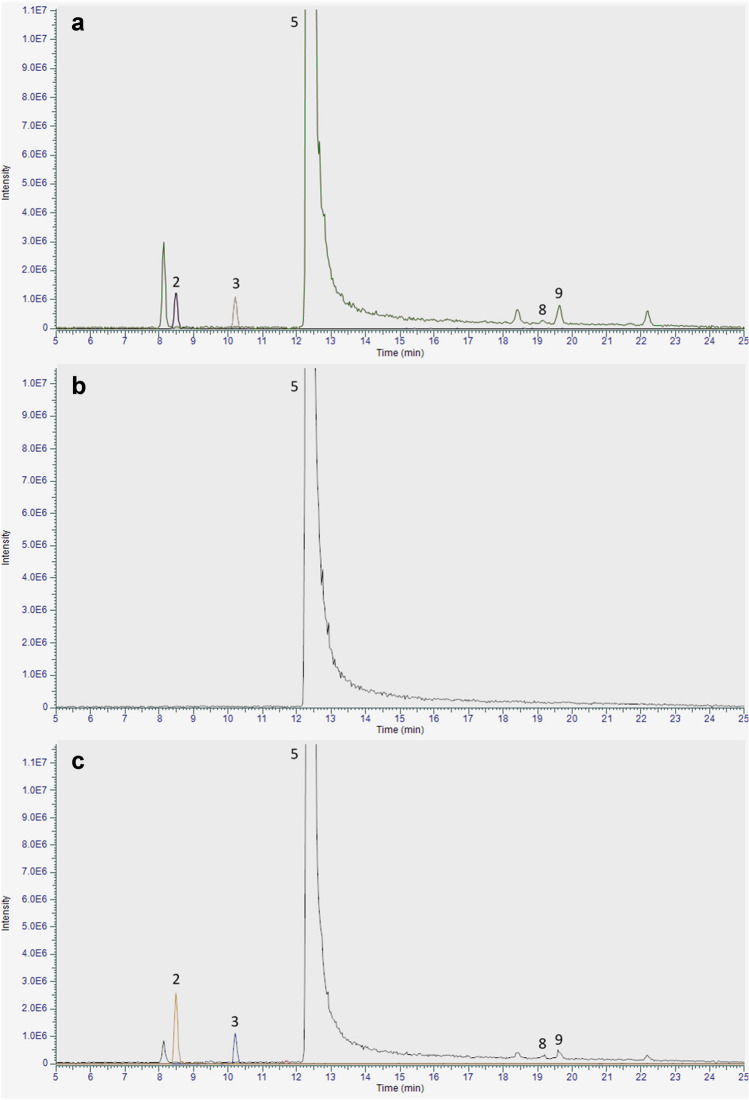
Representative extracted ion chromatograms (EIC) from the UHPLC-HRMS analysis of sample Nat12 (**a**), Syn6 (**b**), and Syn7 (**c**). For peak identification, see Table 1

As for natural CBD samples, CBDV, CBDB, Δ^9^-THC, and Δ^8^-THC were found to be the main impurities detected. CBC was not detected in any of the analyzed samples. A representative EIC from the UHPLC-HRMS analysis of sample Nat12 is shown in Fig. 4a, where it is possible to observe the presence of all the above-mentioned natural impurities.

The UHPLC-HRMS analysis performed on synthetic CBD indicated no impurities among the ones taken into consideration in this study (Fig. 4b), with the only exception of three samples, namely Syn7, Syn8, and Syn9 (Fig. 4c). Indeed, even if they were declared as synthetic compounds, they were found to contain both CBDV and CBDB as the main impurities. Sample Syn7 showed the presence of Δ^9^-THC and Δ^8^-THC as impurities (Fig. 4c), in addition to previously mentioned ones. The presence of compounds usually identified in CBD samples obtained by the extraction of the plant material might suggest an erroneous classification.

### Validation of the HPLC-UV/Vis method

Given the wide possible chances of application of the HPLC-UV/Vis technique in the pharmaceutical analysis of APIs and drug products, the method developed in this work was submitted to validation according to the Q2(R2) guidelines of the International Council for Harmonization of Technical Requirements for Pharmaceuticals for Human Use (ICH) [25].

Linearity, sensitivity, precision, and accuracy were the parameters considered for the method validation.

For what concerns linearity, a five-point calibration curve was built in the concentration range of 2.5–50 µg/mL for impurities identified in natural CBD samples (CBDV, Δ^9^-THC, Δ^8^-THC, CBC, and CBDB). Conversely, the calibration curve for CBD was formulated using a 1.0 mg/mL stock solution. The resulting calibration curve was constructed with five points in the range 51.5–206.0 µg/mL. Correlation coefficient values of the calibration curves were in the range 0.996–0.999 (Table S1, Supplementary Material).

Experimental determination of limit of detection (LOD) and limit of quantification (LOQ) values for each target compound involved subsequent injections of standard solutions at progressively diminishing concentrations of the target compounds until achieving the signal-to-noise (*S*/*N*) criteria for LOD and LOQ (*S*/*N* = 3 and *S*/*N* = 10, respectively). The LOD value ranged from 0.3 to 1.6 µg/mL, while LOQ were in the range 0.8–5.2 µg/mL (Table S1, Supplementary Material).

Precision was assessed intra-day and inter-day by conducting multiple injections of a 50 µg/mL stock solution containing all compounds under investigation in this work, arranged by *t*_R_ as follows: CBGV, CBDV, CBDB, CBG, CBD, abn-CBD, CBCV, Δ^9^-THC, Δ^8^-THC, and CBC. These injections were performed in triplicate over three consecutive days. Relative standard deviation (RSD) data, obtained on both *t*_R_ (< 0.5%) and peak areas (< 9.7%), demonstrated an acceptable precision of the analytical method (Table S2, Supplementary Material).

Accuracy was assessed using the % recovery for both CBD and the impurities considered in this study, including CBDV, CBDB, Δ^9^-THC, Δ^8^-THC, and CBC. To determine the accuracy of the method, 5 mg of both a natural and a synthetic CBD sample were weighed and then dissolved with MeOH to reach a final concentration of 1 mg/mL. These solutions were subsequently diluted to 200 µg/mL with MeOH and injected into the HPLC-UV/Vis system to determine the measured concentration by interpolating the peak area values with the CBD calibration curve. As for CBD impurities, a working solution at 5 µg/mL was prepared by diluting a stock solution at 1000 µg/mL for CBDV, Δ^9^-THC, Δ^8^-THC, and CBC, and at 100 µg/mL for CBDB. All analyses were performed in duplicate for each sample. Accuracy values of target analytes ranged from 82.9 to 106.2%, confirming the reliability of the developed method (Table S3, Supplementary Material).

In general, all the results obtained in the method validation show that the method is compliant with ICH guidelines.

### Purity assessment of CBD samples

Table 2 shows the % relative peak areas of CBD and its impurities in the samples analyzed. Six synthetic CBD samples (Syn1–6) possess a 100% CBD purity, as no other compounds were detected in the chromatograms. Therefore, based on the HPLC data, they were confirmed to derive from organic synthesis and subsequent purification processes.

**Table 2:** Percentage relative peak area data of CBD and its impurities, expressed as mean ± SD (*n* = 4)

Sample	CBGV	CBDV	CBDB	CBG	CBD	abn-CBD	CBCV	Δ^9^-THC	Δ^8^-THC	CBC
Syn1	-	-	-	-	100.0^a^	-	-	-	-	-
Syn2	-	-	-	-	100.0^a^	-	-	-	-	-
Syn3	-	-	-		100.0^a^	-	-	-	-	-
Syn4	-	-	-	-	100.0^a^	-	-	-	-	-
Syn5	-	-	-	-	100.0^a^	-	-	-	-	-
Syn6	-	-	-	-	100.0^a^	-	-	-	-	-
Syn7	-	0.4^a^	0.3^a^	-	99.1±0.1	-	-	-	-	-
Syn8	-	0.6^a^	0.3±0.1	-	99.1±0.1	-	-	-	-	-
Syn9	-	0.2^a^	0.2^a^	-	99.6^a^	-	-	-	-	-
Nat1	-	0.2^a^	0.2^a^	-	99.1±0.1	-	-	0.1^a^	0.1^a^	-
Nat2	-	0.1^a^	0.4^a^	-	99.4^a^	-	-	-	-	-
Nat3	-	0.2^a^	0.2^a^	-	99.6^a^	-	-	-	-	-
Nat4	-	0.4^a^	0.2^a^	-	99.4^a^	-	-	-	-	-
Nat5	-	0.1^a^	0.2^a^	-	99.7^a^	-	-	-	-	-
Nat6	-	0.5±0.1	0.2^a^	-	99.3±0.1	-	-	-	-	-
Nat7	-	0.2^a^	0.3±0.1	-	99.5±0.1	-	-	-	-	-
Nat8	-	0.3^a^	0.3^a^	-	99.0^a^	-	-	0.1^a^	0.1^a^	-
Nat9	-	0.2^a^	0.3^a^	-	99.0±0.1	-	-	0.1^a^	0.1^a^	-
Nat10	-	0.2^a^	0.2^a^	-	99.5^a^	-	-	-	-	-
Nat11	-	0.1^a^	0.3^a^	-	99.4±0.1	-	-	-	-	-
Nat12	-	0.2±0.1	0.3^a^	-	98.8±0.1	-	-	0.1^a^	0.1^a^	-
Nat13	-	0.5±0.1	0.2^a^	-	99.2±0.2	-	-	-	-	-
Nat14	-	0.3^a^	0.3^a^	-	99.1±0.1	-	-	-	-	-
Nat15	-	0.5^a^	0.5^a^	-	97.5^a^	-	-	-	-	-
Nat16	-	0.3^a^	0.3^a^	-	99.3^a^	-	-	-	-	-
Nat17	-	0.1^a^	0.2^a^	-	99.7^a^	-	-	-	-	-
Nat18	-	-	0.2^a^	-	99.7^a^	-	-	-	-	-

^a^SD < 0.05

CBD samples named Syn7, Syn8, and Syn9 exhibited impurities typical of naturally derived CBD, with only CBDV and CBDB quantifiable, according to the criteria previously described. Therefore, a natural origin could be supposed for these samples. This hypothesis was supported also by the high value of CBD purity for samples Syn7 and Syn8 (99.1%) and for Syn9 (99.6%).

All eighteen CBD samples were listed as naturally derived ones, in agreement with the supplier initial classification. The purity values ranged between 97.5 and 99.7%. Sample Nat15 was found to be the one with the lowest purity (97.5%), containing a considerable percentage of CBDV and CBDB impurities (0.5% both), along with other unidentified impurities. Given the greenish color of this sample, the presence of chlorophylls was supposed. The sample Nat12 corresponds to the second one with the lowest purity, with a value of 98.8%, which is justified by the presence of CBDV, CBDB, Δ^9^-THC, and Δ^8^-THC impurities, making it one of the most representative natural samples along with Nat1 (99.1%) and Nat8 and Nat9 (both 99.0%). Nat3, Nat5, Nat17, and Nat18 samples showed a particularly high purity value in the range of 99.6–99.7%.

In general, the main impurities quantified in natural CBD samples were CBDV and CBDB, in a % in the ranges 0.1–0.6% and 0.2–0.5%, respectively.

### Principal component analysis (PCA)

Principal component analysis (PCA) constitutes an exploratory multivariate analysis of a dataset that leverages mathematical principles to improve visualization of the information present in a dataset and to provide a comprehensive overview on the existence of trend, similarity, and/or differences among investigated samples [26]. In the present study, PCA facilitated the visualization of the information by simultaneously considering all the investigated variables. Indeed, it was possible to relate the impurity profile determined for each analyzed sample with its origin, either natural-derived or synthetic, supporting the considerations made following the previous analyses. Additionally, PCA was employed to better visualize the designation of samples Nat3, Nat5, and Nat17, originally declared as natural, and the sample Syn9, initially declared as synthetic, but showing impurities typical of natural CBD. The obtained data were organized into a numerical matrix with dimensions of 52 × 3 (samples with replicates × monitored variables). To standardize the influence of variables on the model, data were auto-scaled (unit variance). The PCA model was constructed using two principal components with an *R*^2^ value of 91.08% of explained variance.

**Fig. 5:**
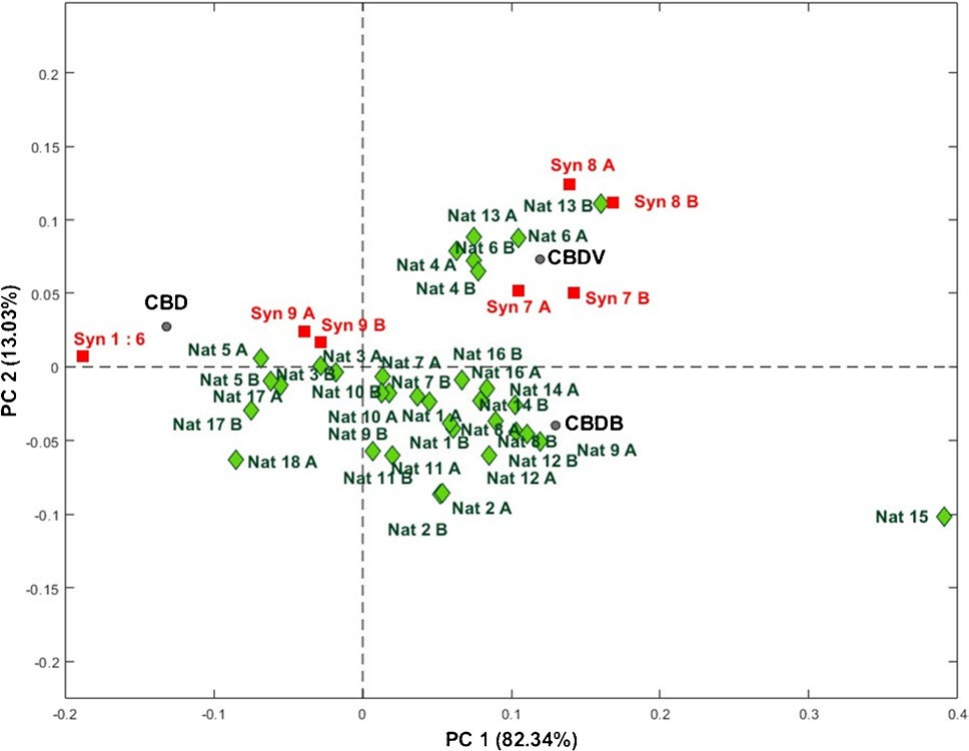
Biplot graph of the first two principal components (PC1 vs. PC2). Synthetic CBD samples (Syn) are depicted in red, while natural CBD samples (Nat) in green. The loading values of the monitored variables are indicated by gray circles (CBDV, CBDB, CBD). Suffixes A and B relate to the first and second sets of sample replicates, respectively

The biplot graph of the first two principal components (PC1 vs. PC2) is shown in Fig. 5, simultaneously representing the scores and loadings obtained from the PCA analysis while maintaining the same scale. Samples originally classified as synthetic (Syn) are depicted with red symbols and labels, while those originally classified as natural (Nat) are represented with green symbols and labels. The loading values of the monitored variables are indicated by gray circles (CBDV, CBDB, CBD). As mentioned in the “Materials and methods” section, preparation was performed in duplicate for each sample; in light of this, the suffix A on the sample relates to the first set of sample preparation, while the suffix B relates to the second set of sample preparation.

An initial assessment of the results reveals a significant reproducibility among the A and B replicates obtained for each investigated sample and a clear distinction of sample Nat15 (characterized by extremely positive PC1 values) from other natural samples. This behavior is attributed to lower values of CBD and higher values of CBDV and CBDB compared with the other natural samples.

Furthermore, among natural CBD samples, an additional subdivision into three groups emerges: samples Nat4, Nat6, and Nat13 exhibit positive values in both principal components and appear to have higher CBDV values; samples Nat3, Nat5, Nat17, and Nat18 show negative values in both principal components with higher CBD values; the other natural CBD samples display positive PC1 values and negative PC2 values, indicating relatively higher CBDB quantities.

For what concerns synthetic CBD samples, a clear difference is observed between Syn7 and Syn8, which exhibit scores very similar to the first group of natural CBD samples. The sample Syn9 aligns closely with the second group of natural samples (high CBD and low CBDV and CBDB values), while all other synthetic samples settle at negative PC1 values (high CBD values).

A more in-depth analysis of the figure suggests that natural samples are primarily distinguished by positive PC1 values, indicating lower CBD content and higher quantities of CBDV and CBDB compared to synthetic samples. However, some samples exhibit different trends, such as Syn7 and Syn8, which are more similar to the composition of natural samples. As observed earlier, Syn7 and Syn8 differ from Syn9 as they present a higher purity value and lower CBDV and CBDB values. Thus, these three CBD samples could be regarded as natural ones.

Samples Nat3, Nat5, Nat17, Nat18, and Syn9 have all negative PC1 score values. These natural samples are closer to the composition of Syn9 and, in general, to synthetic samples, considering the low CBDV and CBDB values and high CBD purity, falling at intermediate values between those found for natural and synthetic samples.

### UHPLC-HRMS analysis of minor impurities

To definitely clarify the origin of CBD samples, a further investigation by UHPLC-HRMS was undertaken focused on minor impurities, including the hexyl and the heptyl homologues of CBD, i.e., CBDH and CBDP, the presence of which can be considered a definite proof of natural origin, being them present in very low amount in the plant material [22, 23]. Since these compounds, when present, are normally found in trace amounts, all CBD samples considered were injected into the UHPLC-HRMS equipment at 1 mg/mL concentration.

**Fig. 6:**
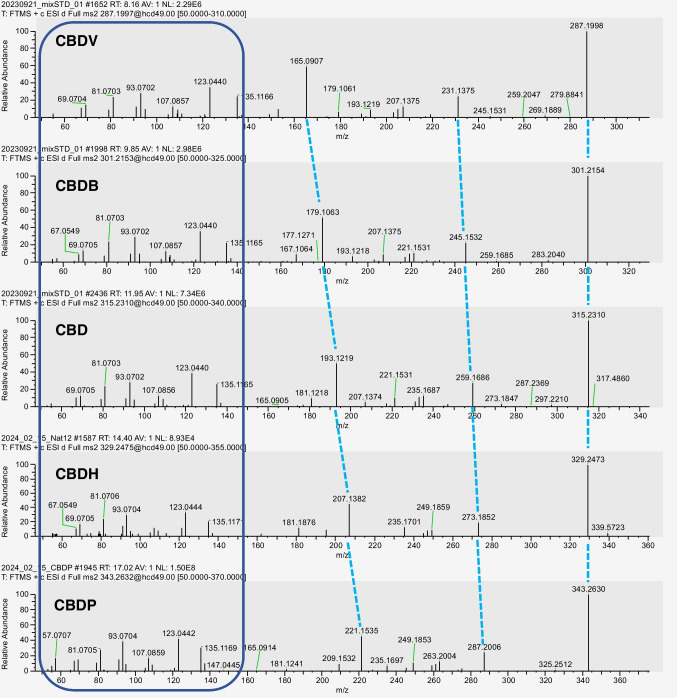
Fragmentation pattern of CBD and structurally related compounds. The blue box includes product ions related to the fragmentation of the terpene moiety, which remain unaltered for each compound. Light blue dashed lines refer to matching product ions among the cannabinoids

The fragmentation of CBD and related compounds is shown in Fig. 6. The UHPLC-HRMS analysis of all natural CBD samples revealed the presence of a peak at 14.4 min with a [M+H]^+^ at *m/z* 329.2473 (Fig. S1, Supplementary Material). The elution time is greater than that of CBD, thus indicating a lower polarity with respect to the latter. The fragmentation pattern of this compound is consistent with that of CBDV, CBDB, and CBD, with the product ions differing only by a methylene unit (Fig. 6). The same was observed for the peak eluting at 19.9 min in the chromatograms, which was found in all CBD samples of natural origin, having a [M+H]^+^ at *m/z* 343.2630 (Fig. S3, Supplementary Material). Also in this case, the fragmentation pattern of the compound matches those of the other belonging to CBD series, with only differences in the product ions due to methylene groups [19]. As it is possible to see in Fig. 6, the product ions at *m*/*z* 273.1852 and 287.2006 in the fragmentation spectra of the peaks at 14.4 and 19.9 min, respectively, have the same ion abundance of the product ions at *m/z* 259.1686, 245.1532, and 231.1375 in the MS/MS spectra of CBD, CBDB, and CBDV, respectively, deriving from the loss of methylene units from the terpene moiety [19]. The product ions at *m/z* 207.1382 and *m/z* 221.1535 of the two compounds correspond to the complete loss of the terpene moiety, except for one carbon unit, and they differ by methylene units from those at *m/z* 193.1219 for CBD, 179.1063 for CBDB, and 165.0907 for CBDV, respectively, with comparable ion abundance. The other minor product ions found in the MS spectra correspond to the fragmentation of the terpene moiety and are the same for all compounds. Fragmentation data were compared to those available in the literature and with those obtained with pure reference compounds [22, 23, 27]. Therefore, the peaks at 14.4 and 19.9 min were attributed to CBDH and CBDP, respectively. These compounds were detected in all natural CBD samples, the only exception being sample Nat18, where only CBDH was detected (Figs. S1 and S3, Supplementary Material). CBDH and CBDP were not detected in any of the synthetic CBD samples, with the only exception of samples Syn2 and Syn7–9 (Figs. S2 and S4, Supplementary Material). As to Syn2, a peak at 17.2 min was observed, but the exact mass did not match that of CBDP. In samples Syn7–9, conversely, both CBDH and CBDP were detected, and their abundance was found to be equal to those observed in natural CBD samples, thus definitely confirming the natural origin of these three samples.

### Enantioselective HPLC analysis

An additional HPLC analysis was carried out to assess the enantiomeric purity of CBD samples and verify whether a difference in the enantiomeric composition of natural and synthetic CBD samples occurs. According to the literature, CBD possesses two stereogenic centers, potentially existing in four stereoisomers: (−)-*trans*-CBD, (+)-*trans*-CBD, (−)-*cis*-CBD, and (+)-*cis*-CBD [28]. Among CBD isomers, the *cis*-CBD form has never been identified before [28]. Therefore, within the samples investigated in this work, only (−)-*trans* and (+)-*trans*-CBD enantiomers were considered. Since in the hemp plant CBDA, which is the acidic precursor of CBD, and, consequently, CBD are selectively biosynthesized in the (−)-*trans* form, the presence of the (+)-*trans*-CBD enantiomer in the analyzed samples could hypothetically indicate a synthetic origin of the sample.

**Fig. 7:**
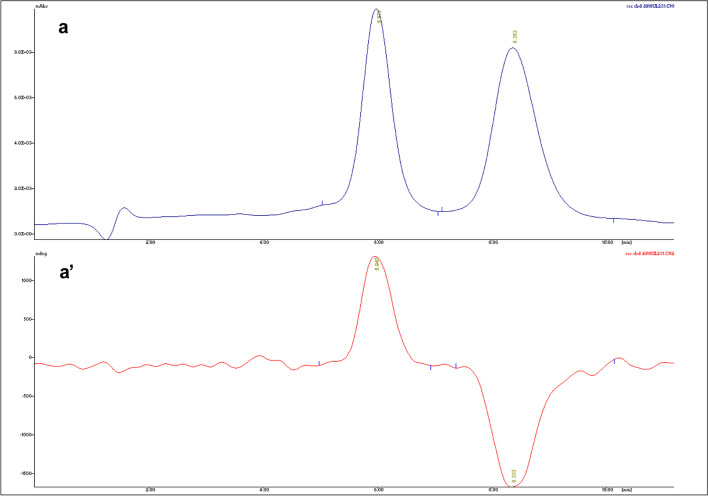
HPLC-UV/Vis (a) and HPLC-CD (a′) chromatograms of a racemic mixture of (+)-*trans* and (−)-*trans*-CBD

Looking at Fig. 7, related to the HPLC-UV/Vis and HPLC-CD analysis of (+)-*trans* and (−)-*trans*-CBD enantiomers, it is possible to observe a good separation of the two analytes with *t*_R_ at 4.5 and 10.0 min, respectively [26]. Moreover, the distinct behavior of the two enantiomers at the CD detector is clear: the different absorbance of the two stereoisomers results in a greater absorption of the right-circularly polarized light for (+)-*trans*-CBD, justifying the acquired positive signal, and greater absorption of left-circularly polarized light for (−)-*trans*-CBD, justifying the negative signal (Fig. 7).

**Fig. 8:**
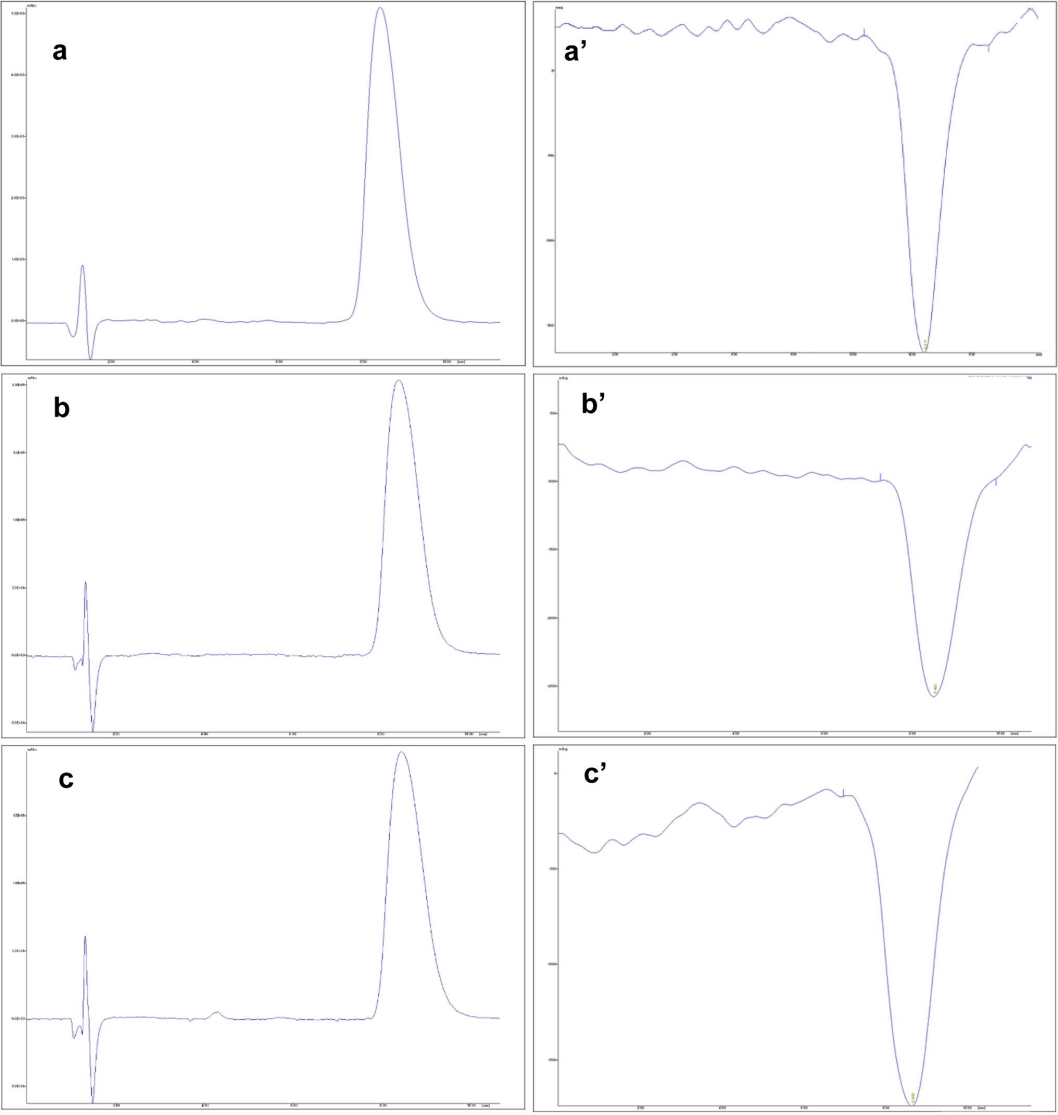
Representative HPLC-UV/Vis (on the right) and HPLC-CD (on the left) chromatograms of samples Nat12 (a, a′), Syn6 (b, b′), and Syn7 (c, c′)

The analyses of CBD samples indicated the presence of the (−)-*trans*-CBD enantiomer only in both natural and synthetic samples, which is consistent with the literature [28]. Representative chromatograms from the HPLC-UV/Vis and HPLC-CD chiral analysis of a natural CBD sample (Nat12), a synthetic CBD sample (Syn6), and a sample declared as synthetic containing impurities characteristic of natural CBD (Syn7) are shown in Fig. 8.

## Conclusions

This study aimed to develop a highly reliable HPLC method with different detectors able to determine CBD purity and establish its origin based on the impurities detected in the samples. Both UHPLC-HRMS and HPLC-UV/Vis were applied for qualitative and semi-quantitative analyses of impurities in solid CBD samples, respectively.

A total of twenty-seven CBD samples were analyzed, including seventeen declared as natural and eight as synthetic. The chromatographic analyses confirmed the origin declared for all the natural samples and for five synthetic samples, with the natural ones showing the characteristic presence of minor cannabinoids derived from the plant matrix (mainly CBDV and CBDB), which are totally absent in synthetic samples. Synthetic samples were characterized by very high CBD purity percentages (100%), while natural CBD exhibited purity ranging from 97.5 to 99.7%. Three synthetic CBD samples (Syn7–9) were found to be of natural origin, as further demonstrated by the analysis of the minor cannabinoids CBDH and CBDP.

To verify whether the natural or synthetic origin of CBD samples can be discriminated taking advantage of different CBD enantiomeric abundance, HPLC-CD analysis was also carried out to evaluate the enantiomeric purity. The result showed the presence of the (−)-*trans*-CBD enantiomer in both synthetic and natural samples, indicating that chiral HPLC does not constitute a discriminating analysis to establish CBD origin.

The overall results of this study obtained by HPLC coupled with HRMS and UV/Vis indicate that the method represents a reliable tool for the characterization of both the purity and the origin of CBD, thus being capable of discriminating if it was obtained either by extraction from hemp inflorescences or by synthesis.

### Supplementary Information

Below is the link to the electronic supplementary material.

Supplementary file1 (DOCX 1990 KB)
