# Supplementary material for: A new HPLC method with multiple detection systems for impurity analysis and discrimination of natural versus synthetic cannabidiol
Source: Anal Bioanal Chem. 2024 Jun 28;416(20):4555–69. doi: 10.1007/s00216-024-05396-5 (PMC11294429; doi:10.1007/s00216-024-05396-5)
Supplement: Supplementary file 1 — Supplementary file1 (DOCX 1990 KB) [file 216_2024_5396_MOESM1_ESM.docx]

**Supplementary Material**

**A new HPLC method with multiple detection systems for impurity analysis and discrimination of natural versus synthetic cannabidiol**

Virginia Brighenti^a^, Matilde Marani^a^, Clarissa Caroli^a,b^, Laura Bertarini^a,b^, Alessio Gaggiotti^c^, Federica Pollastro^d^, Caterina Durante^e^, Giuseppe Cannazza^a^, Federica Pellati^a*^

^a^*Department of Life Sciences, University of Modena and Reggio Emilia, via G. Campi 103, 41125, Modena, Italy*
*^b^Clinical and Experimental Medicine PhD Program, University of Modena and Reggio, Via Giuseppe Campi 287, 41125 Modena, Italy*

*^c^Farmech, Piazza Duomo 20, 20122 Milano, Italy*

*^d^Department of Pharmaceutical Sciences, University of Eastern Piedmont, Largo Donegani 2, 28100 Novara, Italy*

*^e^Department of Chemical and Geological Sciences, University of Modena and Reggio Emilia, Via G. Campi 103, Modena 41125, Italy*

*Corresponding author:

Prof. Federica Pellati

Phone: +39-059-2058565

E-mail: [federica.pellati@unimore.it](mailto:federica.pellati@unimore.it)

| **Table S1**  Linearity ad sensitivity data of the HPLC-UV/Vis method | | | | | | | |
| --- | --- | --- | --- | --- | --- | --- | --- |
| **Compound** | **Concentration range (µg/mL)** | **Correlation coefficient**  **(*r*^2^)** | | | | **LOD (µg/mL)** | **LOQ (µg/mL)** |
| CBDV | 2.5-50.0 | 0.998 |  |  |  | 0.3 | 1.0 |
| CBDB | 2.5-50.0 | 0.998 |  |  |  | 0.6 | 2.1 |
| Δ^9^-THC | 2.5-50.0 | 0.999 |  |  |  | 0.3 | 0.8 |
| Δ^8^-THC | 2.5-50.0 | 0.996 |  |  |  | 1.0 | 3.4 |
| CBC | 1.7-66.5 | 0.998 |  |  |  | 1.6 | 5.2 |
| CBD | 51.5-206.0 | 0.999 |  |  |  | 0.4 | 1.3 |

| **Table S2**  Intra- and inter-day precision of the HPLC-UV/Vis method for the target analytes, expressed as mean of *t*_R_ (min) ± RSD and peak area (mAU**×**s) ± RSD (*n =* 9) | | | | | | | | | | | | | |
| --- | --- | --- | --- | --- | --- | --- | --- | --- | --- | --- | --- | --- | --- |
| **Compound** | **Day 1** | |  | | **Day 2** | |  | **Day 3** | |  |  | **Inter-day** |  |
|  | ***t*_R_**  **(min)** | **Area**  **(mAU×s)** | | ***t*_R_**  **(min)** | | **Area**  **(mAU×s)** | | ***t*_R_**  **(min)** | **Area**  **(mAU×s)** |  | ***t*_R_**  **(min)** |  | **Area**  **(mAU×s)** |
| CBGV | 8.5±0.5 | 3750.9±3.3 | | 8.5±0.1 | | 3845.0±4.3 | | 8.5±0.1 | 3991.9±8.8 |  | 8.5±0.2 |  | 3862.6±5.9 |
| CBDV | 8.7±0.3 | 8431.9±4.5 | | 8.7±0.1 | | 9069.5±9.3 | | 8.7±0.1 | 9186.6±6.7 |  | 8.7±0.2 |  | 8896.0±7.4 |
| CBDB | 10.5±0.2 | 2627.7±5.7 | | 10.5±0.1 | | 2781.4±5.2 | | 10.5±0.1 | 2834.8±6.3 |  | 10.5±0.2 |  | 2747.9±6.0 |
| CBG | 12.1±0.2 | 5374.6±6.8 | | 12.1±0.1 | | 5661.7±5.7 | | 12.1±0.1 | 5752.9±5.7 |  | 12.1±0.1 |  | 5596.4±6.1 |
| CBD | 12.8±0.2 | 5976.9±4.8 | | 12.8±0.1 | | 6343.3±5.0 | | 12.8±0.1 | 6515.2±6.5 |  | 12.8±0.1 |  | 6278.5±6.1 |
| abn-CBD | 15.6±0.1 | 5007.0±4.6 | | 15.6^a^ | | 5334.9±5.6 | | 15.6±0.1 | 5403.6±6.5 |  | 15.6±0.1 |  | 5284.5±6.1 |
| CBCV | 16.4±0.1 | 1305.1±3.6 | | 16.4^a^ | | 1345.7±9.7 | | 16.4±0.1 | 1377.2±8.4 |  | 16.4±0.1 |  | 1342.7±7.1 |
| Δ^9^-THC | 20.4±0.1 | 7193.7±5.0 | | 20.5^a^ | | 7614.6±5.4 | | 20.5±0.1 | 7735.1±5.4 |  | 20.5±0.1 |  | 7514.5±5.6 |
| Δ^8^-THC | 20.9±0.1 | 2522.6±1.5 | | 21.0^a^ | | 2736.7±4.2 | | 21.0±0.1 | 2788.0±7.0 |  | 21.0±0.1 |  | 2682.4±6.2 |
| CBC | 23.2±0.1 | 967.9±5.2 |  | | 23.3^a^ | 23.3±0.1 |  | 23.3±0.1 | 1043.0±5.8 |  | 23.3±0.1 |  | 1000.1±6.0 |
| ^a^ RSD < 0.05 | | | | | | | | | | | | | |

| **Table S3**  Accuracy data for the target compounds, expressed as % recovery ± RSD | |
| --- | --- |
| **Compound** | **% Recovery** |
| CBDV | 93.9±4.5 |
| CBDB | 94.0±3.9 |
| Δ^9^-THC | 82.9±1.4 |
| Δ^8^-THC | 96.6 ^a^ |
| CBC | 91.9±0.1 |
| CBD (synthetic) | 104.0±2.6 |
| CBD (natural) | 106.2±0.4 |
| ^a^ RSD < 0.05 | |


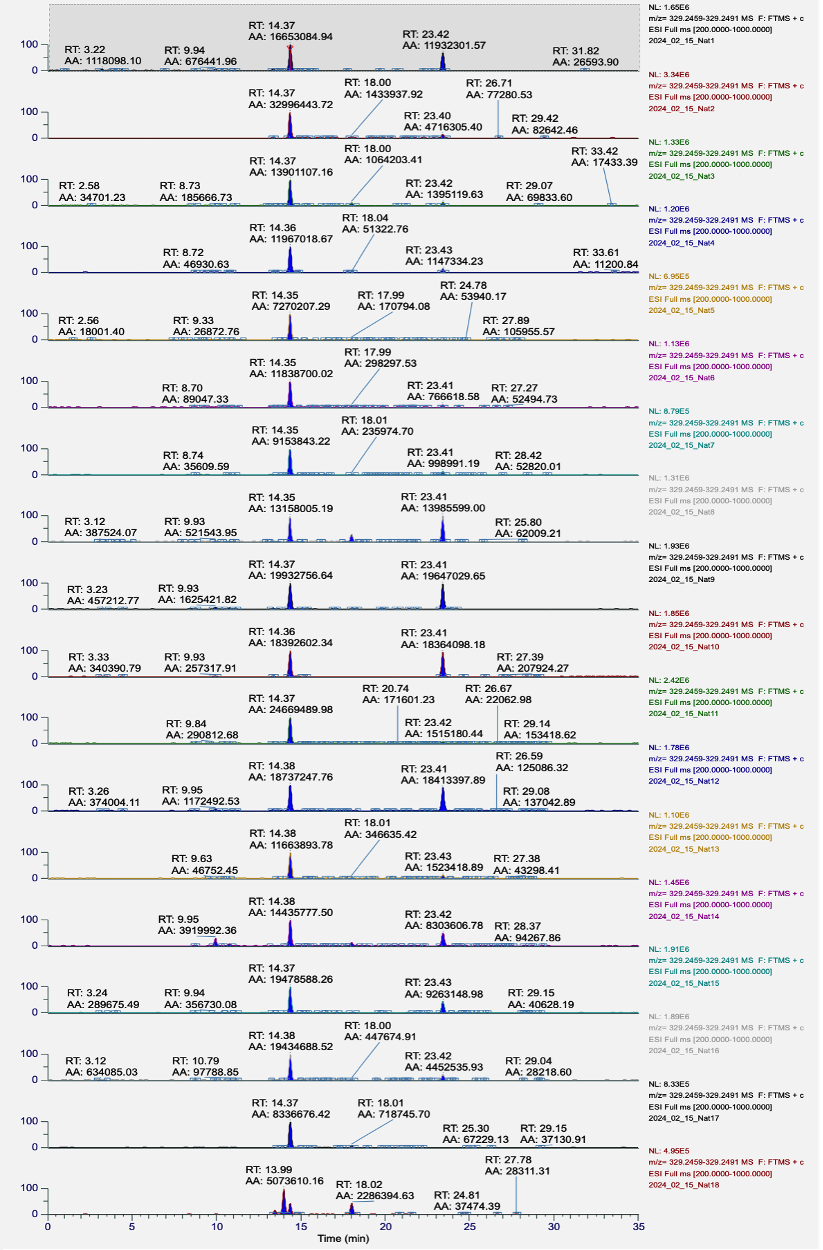


**Fig. S1** Extracted Ion Chromatograms (EIC) of CBDH in samples of natural CBD. Area of the peaks were obtained by extracting the exact mass (mass tolerance: 5 ppm) of CBDH ([M+H]^+^ = 329.2473).


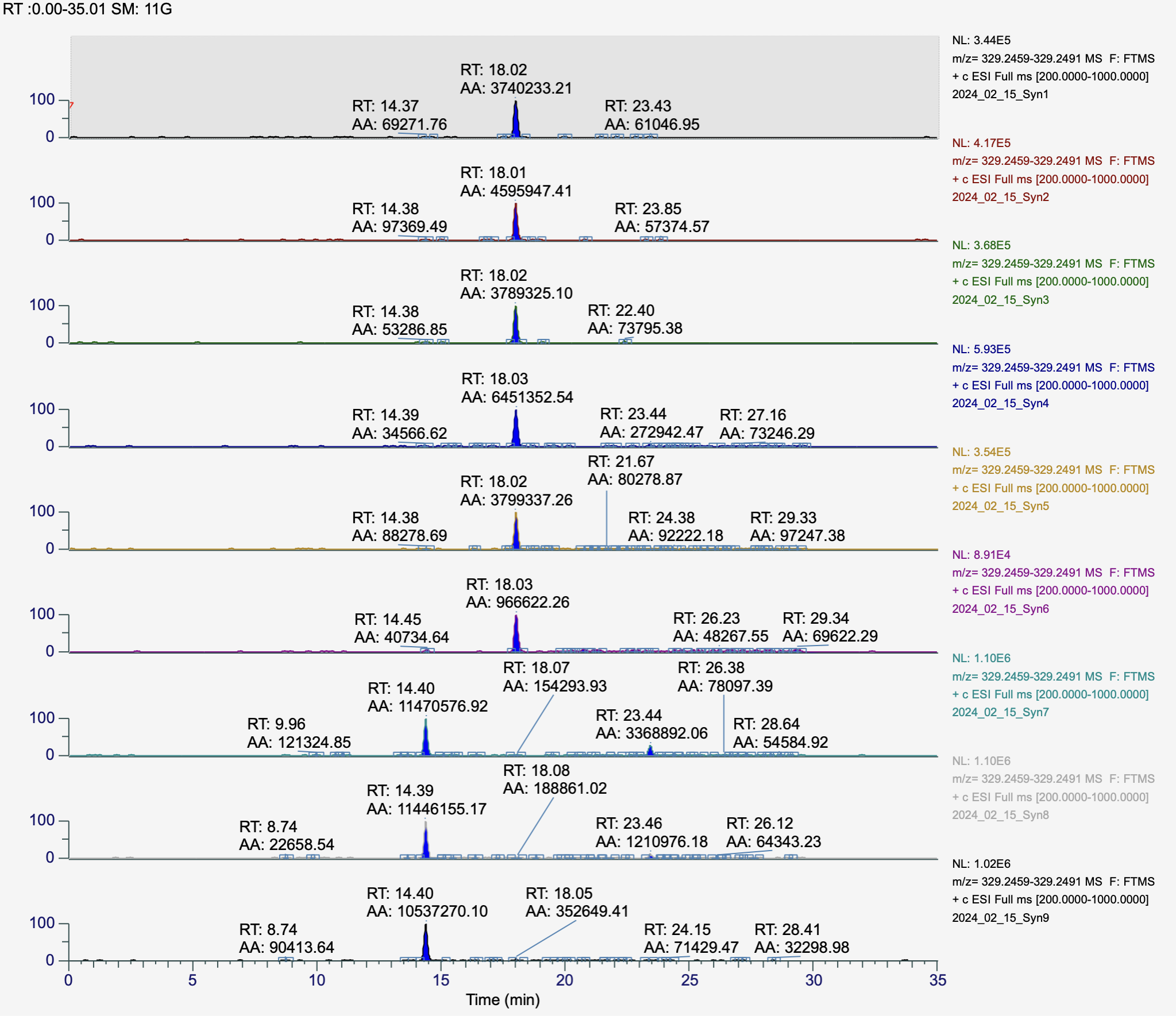


**Fig. S2** Extracted Ion Chromatograms (EIC) of CBDH in samples of synthetic CBD. Area of the peaks were obtained by extracting the exact mass (mass tolerance: 5 ppm) of CBDH ([M+H]^+^ = 329.2473).


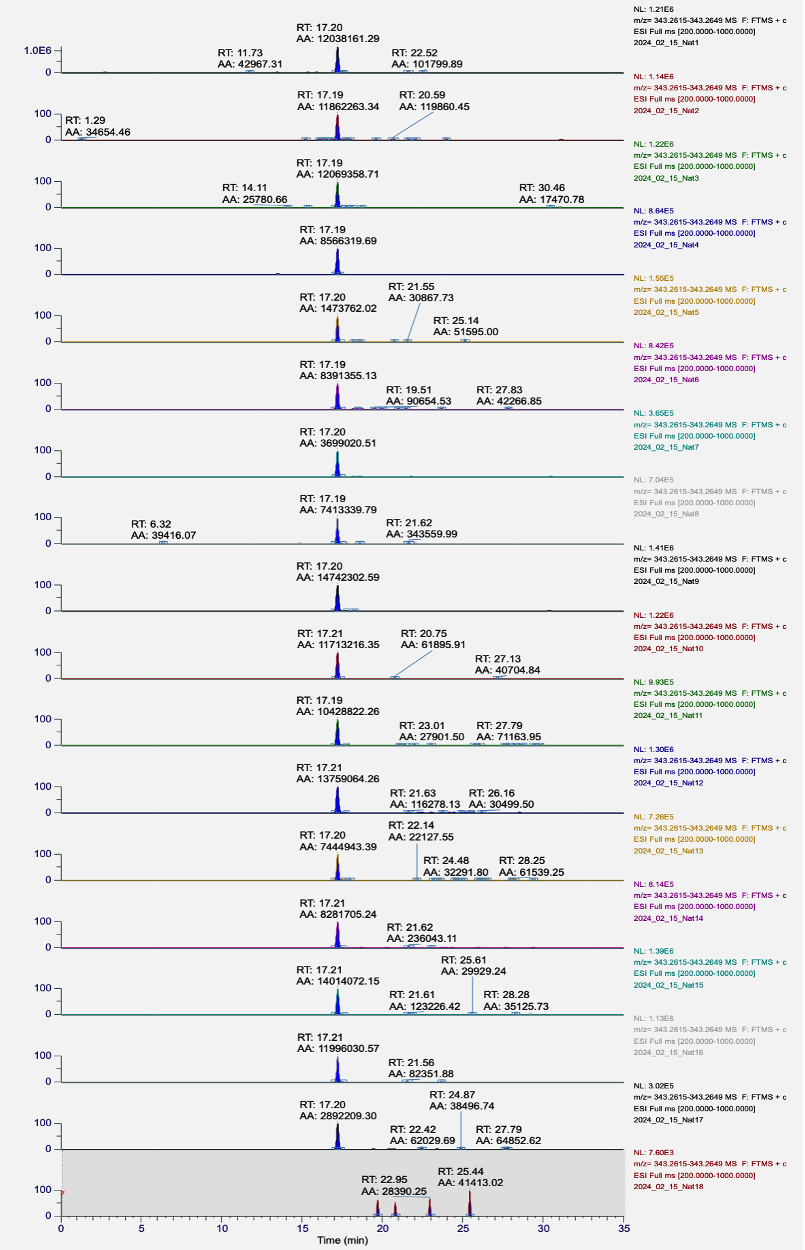


**Fig. S3** Extracted Ion Chromatograms (EIC) of CBDP in samples of natural CBD. Area of the peaks were obtained by extracting the exact mass (mass tolerance: 5 ppm) of CBDP ([M+H]^+^ = 343.2630).


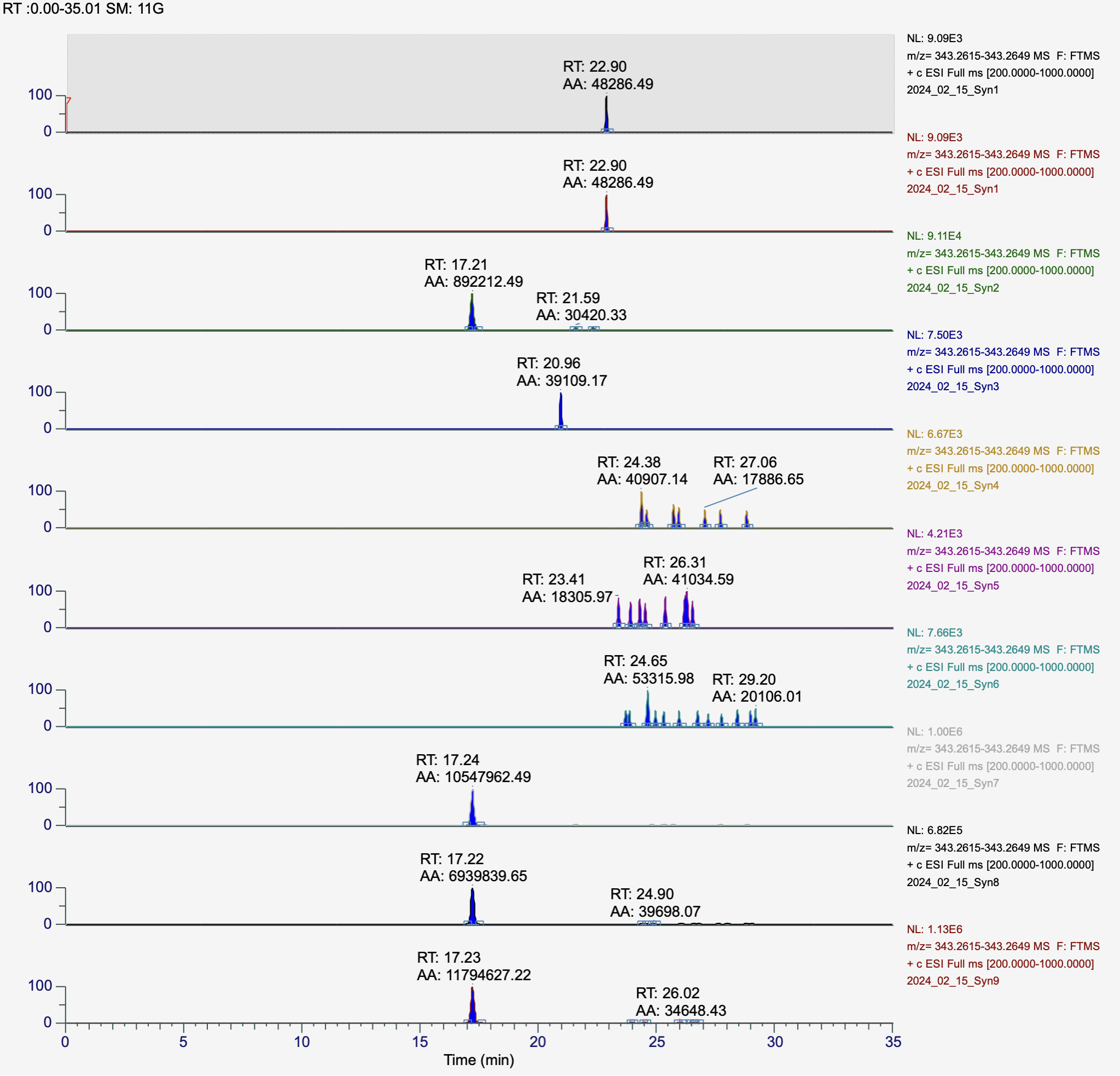


**Fig. S4** Extracted Ion Chromatograms (EIC) of CBDP in samples of synthetic CBD. Area of the peaks were obtained by extracting the exact mass (mass tolerance: 5 ppm) of CBDP ([M+H]^+^ = 343.2630).
